# Audiovestibular Function Deficits in Vestibular Schwannoma

**DOI:** 10.1155/2016/4980562

**Published:** 2016-09-22

**Authors:** Constantin von Kirschbaum, Robert Gürkov

**Affiliations:** Department of Otorhinolaryngology and Head and Neck Surgery, Grosshadern Medical Center, University of Munich, Marchioninistr. 15, 81377 Munich, Germany

## Abstract

*Introduction*. Vestibular schwannomas (VS) are benign tumours of the vestibular nerve and can lead to hearing loss, tinnitus, vertigo, facial palsy, and brainstem compression. Audiovestibular diagnostic tests are essential for detection and treatment planning.* Methods*. Medline was used to perform a systematic literature review with regard to how audiovestibular test parameters correlate with symptoms, tumour size, and tumour location.* Results*. The auditory brainstem response can be used to diagnose retrocochlear lesions caused by VS. Since hearing loss correlates poorly with tumour size, a retrocochlear lesion is probably not the only cause for hearing loss. Also cochlear mechanisms seem to play a role. This can be revealed by abnormal otoacoustic emissions, despite normal ABR and new MRI techniques which have demonstrated endolymphatic hydrops of the inner ear. Caloric and head impulse tests show frequency specific dynamics and vestibular evoked myogenic potentials may help to identify the location of the tumour regarding the involved nerve parts.* Conclusion*. In order to preserve audiovestibular function in VS, it is important to stop the growth of the tumour and to avoid degenerative changes in the inner ear. A detailed neurotological workup helps to diagnose VS of all sizes and can also provide useful prognostic information.

## 1. Introduction

Vestibular schwannoma (VS) can lead to sensorineural hearing loss, tinnitus, and vertigo. Patients with asymmetric hearing loss, unilateral tinnitus, or vertigo should be evaluated for the presence of a VS.

VS are benign tumours arising from the sheath of the vestibular nerve. They cause dysfunction of the nerves by compressing the nerve fibre and obstructing the blood supply to the nerves [1, 2]. VS may present with a variety of clinical features. Hearing loss is the most common first symptom while vertigo is often the most distressing symptom for the patient. However, hearing loss may also be absent in case of an extrametal growth pattern.

The estimated annual incidence of these tumours ranges from approximately 0.6 to 1.9 per 100,000 [3].

Currently there exist several classifications of VS, based on the tumour size and location.  Table 1 gives a summary of the two most common classifications according to Koos and Samii.

Audiovestibular function tests, such as pure tone audiometry (PTA), otoacoustic emissions (OAEs), auditory brainstem response (ABR), caloric irrigation, head impulse test (HIT), and vestibular evoked myogenic potentials (cVEMPs and oVEMPs) are important for initial diagnosis as well as subsequent monitoring of disease progression.

The current gold standard of diagnosis is MR imaging, but because MRI scans are expensive, they are not universally utilized to screen all patients with audiovestibular symptoms [4]. Thus, VS may be misdiagnosed as a peripheral disease, such as noise-induced hearing loss, presbyacusis, tinnitus, vestibulopathy, Menière's disease, or sudden deafness with vertigo.

Furthermore, precise preoperative audiovestibular assessment can have implications on the decision of the therapeutic approach such as wait and scan, radiotherapy, or microsurgery.

## 2. Methods

English and German articles published between 1972 and 2016 and available on Medline were reviewed in order to produce a summary of the current knowledge with regard to audiovestibular functional changes in patients with VS as diagnosed by MR imaging.

## 3. Results

### 3.1. Audiological Function Tests

#### 3.1.1. Vestibular Schwannomas and Audiometry

Sudden sensorineural hearing loss may be an initial symptom of a VS [5]; therefore, audiometry has been regarded as an initial step in the diagnostic workup for VS.

It has been shown that VS has been diagnosed in 0.8–47.5% of patients with sudden sensorineural hearing loss [6, 7].

To investigate predictive factors or symptoms of future hearing loss Jethanamest et al. retrospectively reviewed patients between 2002 and 2013 who chose observation for their initial management. The estimated median time to loss of serviceable hearing was 6.3 years. No symptoms or factors were identified to be predictive of future hearing loss. However, symptoms of disequilibrium or imbalance appeared to be associated with an increased risk of subsequent tumour growth [8].

Wagner et al. found that the degree of hearing loss before treatment is significantly influenced by the age of the patient (*p* < 0.001) but not by tumour size. At follow-up examinations after treatment with microsurgery and radiotherapy, hearing was significantly worse in those patients with a large VS. Age was not a significant determinant for loss of hearing on the follow-up visits (*p* < 0.08) [9].

Another study noted that 45% of those patients who initially had a hearing ability of AAO class A on the tumour affected side lost class A hearing after 5 years [10].

Concerning speech discrimination, Stangerup et al. demonstrated during a 33-year period that 59% of the patients with a speech discrimination better than 70% preserved good hearing after a mean of 4.7 years of observation. 69% of the patients with a speech discrimination score of 100% at diagnosis maintained good hearing after more than 10 years. Of the patients with only a small discrimination loss at diagnosis, 38% maintained good hearing [11].

Tumour growth less than or equal to 2.5 millimeters per year has been shown to lead to higher rates of hearing preservation. For patients with a small tumour and normal speech discrimination, the main indication for active treatment should be the established tumour growth [11, 12].

Day et al. found in a total of 44 patients that normal hearing or low-frequency hearing loss was correlated with small tumour size. Those with mid- or high-frequency hearing loss had a medium-sized tumour, while those with global frequency hearing loss or total deafness had tumour size larger than 2.5 cm. The authors explained the hearing loss by tumour compression of the cochlear nerve, since most VS originate from the vestibular nerve [13]. This association of small tumour size with low-frequency hearing loss may suggest the presence of endolymphatic hydrops in some of these patients, since hydrops is typically associated with low-frequency hearing loss.

Concerning primary inner ear schwannomas (PIES), that is, schwannomas arising from the labyrinth, a systematic review of 243 patients diagnosed from 1933 to 2011 showed that unilateral hearing loss was the most frequent presenting symptom (99%). Vertigo and abnormal balance were more common among tumours involving the vestibular system (*p* < 0.01) [14]. Positional vestibular symptoms and mixed hearing loss occur especially in intralabyrinthine schwannomas and are suggested to result from the tumour's mass effect within the labyrinth [15]. Especially in intralabyrinthine schwannoma a diagnostic delay of up to 15 years has been reported. Since radiographic findings in MRI may be very subtle, intralabyrinthine schwannomas can easily be overlooked, so that they are frequently diagnosed only years after the onset of symptoms [16].

Overall, there is limited data on the long-term auditory symptoms in patients with sporadic small- and medium-sized VS. The initial treatment strategy for VS is being discussed controversially. The overall prognosis for hearing in sporadic VS is poor regardless of treatment strategy. Good baseline hearing proved to be a strong predictor for maintained serviceable hearing. Observation was associated with the highest rate of hearing preservation [17], although this might be due to a selection bias.

#### 3.1.2. Vestibular Schwannomas and ABR

After audiometry, the next diagnostic test of choice for patients with clinical suspicion of a VS (unilateral or asymmetrical sensorineural hearing loss) was the ABR. However, with improvements in MRI diagnostics in the late 1980s, the usefulness of the ABR in the diagnostic workup of VS has diminished and MRI is nowadays widely considered to be the gold standard for diagnosing retrocochlear pathology.

Currently, the role of the ABR remains controversial. The criteria for assessing abnormal responses on ABR tests have varied considerably among studies. Some authors argue that the ABR has no place in today's management and that MRI should be the initial screening test when there is a suspicion of VS [18]. Other authors argue that there is still a place for the ABR in the diagnostic algorithm due to the high cost and lower availability of MRI [19].

The ABR has a high dependency on the tumour size and the diagnostic sensitivity is especially low for smaller tumours [20].

This was also shown in a study by Koos et al. in which multiple online databases were assessed in order to evaluate the usefulness of ABR tests in 3314 patients from 1978 to 2009. The inclusion criterion was the presence of a surgically or radiographically confirmed VS. The pooled sensitivity of ABR was 93.4%, 85.8% for tumours < 1 cm, and 95.6% for tumours > 1 cm. Moreover, the sensitivity of ABR was higher for extracanalicular than for intracanalicular tumours. The authors concluded that for patients with lower clinical suspicion of a VS an ABR can still provide valuable additional information on which the decision to obtain an MRI can be based.

However, a small number of tumours with audiometrically documented hearing loss demonstrate a normal ABR [21], which supports the hypothesis that schwannomas can also cause hearing loss by purely cochlear mechanisms.

#### 3.1.3. Vestibular Schwannomas and Otoacoustic Emissions

OAEs are active mechanical responses from the cochlea which provide information about the integrity of the preneural cochlear receptor mechanisms. Clinical experience has shown that OAEs are often absent or compromised in VS. In order to objectify the effects of retrocochlear disease on distortion-product otoacoustic emissions (DPOAEs) Telishi et al. classified the DPOAE patterns as cochlear or noncochlear. In a large series of patients with unilateral VS they showed that the majority of ears with tumours demonstrated cochlear (57%), rather than noncochlear (41%) patterns of DPOAEs [22].

The occurrence of cochlear pathology has implications regarding the accuracy of OAEs as a diagnostic tool for VS. If one assumes that VS affect the retrocochlear function (hearing impairment is purely neural in origin) and have little effect on the cochlea itself then VS patients should have preserved OAEs. However, it seems that amplitudes of DPOAEs begin to decrease already at the early stages of hearing loss in VS patients. Comparing a group of VS patients with normal/symmetrical hearing and a group of VS patients with mild hearing loss (threshold at any tested frequency better than 45 dB) on the tumour ear side, Gouveris et al. showed that DPOAE amplitudes do not differ strongly between the ears in VS patients with normal/symmetrical hearing. But DPOAEs are decreased compared with the nontumour ear in VS patients with even mild hearing loss. The tumour size did not differ significantly between these two groups, which suggests a cochlear origin of early hearing loss in these patients. The authors conclude that DPOAEs may be used in a clinical setting to monitor progression of cochlear damage at the early stages of hearing impairment in VS patients [23].

### 3.2. Vestibular Function Tests

#### 3.2.1. Vestibular Schwannomas and Caloric Response

Vertigo has been reported to be one of the risk factors for the growth of VS [26]. Therefore, vestibular function tests such as the caloric test should be used routinely in the workup of VS. By using videonystagmography (VNG) or electronystagmography (ENG), caloric responses can be observed and quantified in terms of slow-phase nystagmus velocities generated during warm and cold irrigations of each ear. The asymmetry between the two horizontal semicircular canals is usually calculated by the Jongkees formula. When unilateral weakness (UW) is less than 25%, the caloric response is regarded as normal.

With the caloric test it is possible to unilaterally stimulate the horizontal semicircular canal, which is innervated by the superior part of the vestibular nerve. Thus, one might think that the caloric response is only significant when the superior branch of the vestibular nerve is affected by the VS. Borgmann et al. tested whether ENG results could predict the nerve of origin before surgery. They defined pathologic caloric test findings as an indicator for superior vestibular nerve schwannomas (SVN) involvement and a normal caloric response as a sign of inferior vestibular nerve schwannomas (IVN) origin. As a result, the nerve of origin could be predicted in 90 of 111 patients (81%), which means that pathologic results in preoperative ENG were significantly more frequent in patients with SVN schwannomas. They concluded that the caloric test helps to predict the nerve of origin of a VS and can be used indirectly as a prognostic factor for hearing preservation because hearing loss due to surgery was significantly lower in patients with tumours of the SVN [27].

In contrast Ushio et al. demonstrated in a study of 109 VS patients that the percentage of abnormal responses in caloric tests was not different between patients with SVN and those with IVN [2]. These results showed no clear correlation between the results of caloric tests and the nerve origin of the tumour.

Tringali et al. observed a good correlation between caloric weakness and tumour size when they tested the preoperative response of 629 tumour patients. In the group of patients with UW > 70%, who had a larger tumour size, postoperative facial palsy was more frequent. Postoperative hearing preservation was more frequently observed in the “normal group” with a UW < 20%. They concluded that a normal caloric response can be a good predictive factor for hearing preservation and normal postoperative facial function [28].

In a prospective pilot study on patients with VS, Wagner et al. compared groups with VS < 20 mm and ≥ 20 mm. In group 1, the median loss of vestibular function was +10.5%; in group 2 (with a tumour size ≥ 20 mm) a higher degree of loss of vestibular function (median UW 36%) was found. Treatment by micro- and radiosurgery caused a further decrease of vestibular function in both groups. So vestibular function clearly correlated with the size of the tumour and also with the method of treatment used. The majority of patients complained about vertigo before and after treatment [9].

Ushio et al. also found that the mean tumour size in patients showing abnormal responses in caloric testing was larger than that in patients showing normal responses. This tendency was not observed for patients with VS within the internal acoustic canal [2]. The same observation was made by Suzuki in patients with unilateral VS histologically diagnosed by surgery. There was no significant difference between patients with normal caloric responses and those with canal paresis with respect to the size of the tumour measured for intracanalicular tumours [29].

On the contrary, the results of a study by Teggi et al. showed that vestibular function is influenced by intracanalicular length and diameter of the tumour, rather than by total tumour volume. The subgroup of 30 patients with a greater intracanalicular length of the tumour presented a higher value of UW than the subgroup with a smaller length [30].

In summary, the available data suggest that the severity of the UW correlates with degree of infiltration of the cochleovestibular nerve by the tumour. This means that an abnormal caloric response is a prognostic factor for hearing preservation surgery [31].

#### 3.2.2. Vestibular Schwannomas and Head Impulse Test

Both the caloric irrigation and the head impulse test (HIT) serve to evaluate the horizontal vestibuloocular reflex (VOR). The HIT applies high-acceleration small-amplitude head impulses around an earth-vertical axis while the patient is fixating a stationary target. A catch-up saccade is observed if the eye no longer compensates for the head movement and accordingly this test is classified as pathologic. The probability of a pathologic HIT generally increases with increasing UW of caloric examination. For patients with a UW within normal limits, a pathological HIT is very unlikely [32].

At present the HIT is the only bedside examination that enables the identification of the side of a unilateral hypofunction of the peripheral vestibular system.

However, a VOR deficit may not be diagnosed because corrective saccades cannot always be detected by simple observation. Besides the manual HIT as described by Halmagyi et al. [33], which can be performed quickly as a bedside test, there is the video head impulse test (vHIT). This allows the identification not only of overt saccades but also of covert saccades occurring during the head movement which are not visible to the naked eye.

2013 Blödow et al. examined 142 patients with acute or chronic vestibular syndrome and found that 47.6% had a pathological vHIT whereas 52.4% had a normal test result. Covert catch-up saccades could be demonstrated in 13.7% whereas in 86.3% overt catch-up saccades alone or in combination with covert catch-up saccades were found. The authors concluded that the vHIT is superior to the clinical HIT because in approximately one in eight cases with acute or chronic peripheral vestibular syndrome a covert catch-up saccade can be detected, which would otherwise have been undetectable by the clinical HIT [34].

The same group recently examined 46 patients with VS and showed that caloric irrigation exhibits a higher sensitivity than HIT (72% versus 41%) and both tests show only a moderate correlation. Tumour size and hearing level were significantly correlated with caloric abnormalities but not with HIT findings [35].

The finding of a selective impairment of the VOR in the low-frequency range (caloric) was also shown in a case of an intralabyrinthine schwannoma, whereas the high-frequency VOR (HIT) remains intact [36]. This constellation is typical for Menière's disease.

However, neither study could find an explanation for the higher sensitivity of the caloric test. Both tests measure the VOR but at different temporal frequencies: the HIT with short head impulses tests high frequencies up to 5 Hz [37] and the caloric irrigation tests lower frequencies around 0.003 Hz [33].

Vestibular testing at different frequencies provides deeper insights into VOR function and can help in detecting cerebellopontine lesions. According to the current literature, both tests have to be considered complementary and are valuable for both diagnostic and therapeutic decisions.

The HIT and the bithermal caloric irrigation are different in terms of not only temporal frequency, but also the method of stimulation. The HIT causes a physiologic endolymphatic flow as a consequence of rapid head impulses. In contrast, caloric irrigation induces endolymphatic flow due to a temperature gradient from one side of the canal to the other. Caloric irrigation additionally stimulates the inner ear in a non-gravity-dependent way [38].

It still remains unclear whether the dissociation of the caloric test and the HIT in patients with VS is due to the varying temporal frequencies or the differing methods of stimulation. Of note, the predilection for caloric test abnormality is typical for patients with Menière's disease (as opposed to, e.g., vestibular neuritis which typically affects both tests) and has been shown to correlate with the degree of endolymphatic hydrops herniation into the horizontal semicircular canal. Therefore, this feature might suggest endolymphatic hydrops in some of these patients.

#### 3.2.3. Vestibular Schwannomas and VEMPs

VEMPs provide information on otolith organ function. The conventional method for recording VEMPs involves measuring electromyographic activity from surface electrodes placed over the tonically activated sternocleidomastoid muscles. The cervical VEMP (cVEMP) is a manifestation of the vestibulocollic reflex [39]. VEMPs can also be recorded from the extraocular muscles using surface electrodes placed near the eyes. These ocular VEMPs (oVEMPs) are a manifestation of the vestibuloocular reflex [40]. Stimulation of the vestibular system is possible using air-conducted sound (ACS) and bone-conducted vibration (BCV).

In recent years the role of cVEMPs and oVEMPs in the assessment of patients with VS has gained increased attention. A number of studies have demonstrated that VEMPs have an important clinical value in the diagnosis of VS because sometimes an abnormal VEMP result may be the only sign of a unilateral VS (the caloric and hearing test being normal) [41].

Physiological and clinical studies have shown that cVEMPs to ACS mainly reflect the function of saccular afferents and the inferior vestibular pathway [42–44].

On the contrary, clinical studies in patients with unilateral VS [45, 46] and animal studies using guinea pigs [47] have suggested that oVEMPs to BCV are likely to reflect the function of the utricle and superior vestibular pathway.

Recently VEMPs have been applied especially for classifying VS according to the involved nerves prior to surgery or radiotherapy.

Iwasaki et al. postulated that BCV oVEMPs mainly reflect the function of the SVN. They hypothesised that if the oVEMPs to BCV mainly reflect the function of the utricular afferents, the results should mostly coincide with those of the caloric test rather than with cVEMPs to ACS. Among ten patients with inconsistent results in these three vestibular tests, seven (70%) showed corresponding results between oVEMPs to BCV and the caloric test, whereas only one patient (10%) showed correspondence between oVEMPs to BCV and cVEMPs to ACS [46], thus supporting the authors' hypothesis.

In a bigger cohort of 45 patients with untreated unilateral VS, the results of oVEMPs to ACS had a significant correlation with those of oVEMPs to BCV. These findings support the hypothesis that oVEMPs in response to both ACS and BCV are predominantly mediated by the superior vestibular nerve and probably both reflect the function of the utricle [48].

However, according to other studies the nerve of origin of tumours cannot be predicted based on VEMPs. In a study of 130 patients histologically diagnosed by surgery, VEMPs in patients with tumours arising from the SVN were not significantly different from those in patients with tumours of the IVN [29]. Ushio et al. also could not find a clear correlation of VEMP results with the nerve origin of the tumour. The percentage of patients showing abnormal responses was not different between 37 patients with VS of the SVN and 26 patients with VS of the IVN. Also, no difference was observed for patients with VS within the internal acoustic canal. The authors hypothesised that large VS affect functions of both the SVN and the IVN, irrespective of the nerve origin, because the space of the internal acoustic canal is limited [2]. As a result tumours injure or compress both parts of the vestibular nerve as they grow. Complicating matters even further, it is likely that these efforts to predict the affected nerve can also be obscured by diffuse labyrinthine damage.

With regard to tumour size, several authors describe that, in VS, ACS cVEMPs are absent or decreased in amplitude in up to 80% of cases [49–51]. The amplitude decreases in association with an increase in tumour size. Larger tumours and those located more medially are more commonly associated with cVEMP abnormalities [13, 52].

Tumour compression of the brainstem and the vestibular spinal tract and compression of the myelin sheath of the IVN, resulting in demyelination of the nerve, were also correlated with cVEMP latencies [29, 46].

However, comparing the tumour size within the internal acoustic canal, Ushio et al. could not observe a difference between patients showing abnormal VEMP responses and patients showing normal responses [2].

In summary, there exists at the moment no consensus about the use of VEMPS in detecting a VS. So it seems currently not possible to make any diagnostic prognosis through VEMPS.

### 3.3. What Could Be the Most Probable Cause of Symptoms Like Hearing Loss or Vertigo in Patients with VS?

Since it is not yet clear whether there are symptoms or parameters (such as tumour growth) which can be used to predict future hearing outcomes in VS patients, one might raise the question whether the hearing loss is caused by retrocochlear or cochlear mechanisms. Clinical and histological observations have suggested that the hearing loss in VS does not correlate with the size of the VS [53, 54], indicating that compression of the cochlear nerve within the internal auditory canal may not be the only mechanism in hearing loss. In other words, it may be that the commonly observed pure tone hearing loss patterns in patients with VS may be caused by cochlear, rather than exclusively retrocochlear mechanisms.

Roosli et al. performed a detailed assessment of cochlear pathology in patients with VS and also found a lack of correlation between tumour size and hearing loss. In a retrospective analysis of temporal bone histopathology they found that neural mechanisms caused by compression of the cochlear nerve are not solely responsible for hearing loss in patients with VS. When compared to the unaffected ear, VS caused significantly more inner and outer hair cell loss and cochlear neuronal loss, precipitate in endolymph and perilymph, and increased pure tone average thresholds. Moreover, the tumour distance from the cochlea and the nerve of origin did not correlate with structural changes in the cochlea or the hearing threshold. Direct invasion of the modiolus or the cochlea by VS was not observed in any case [55].

**Figure 1:**
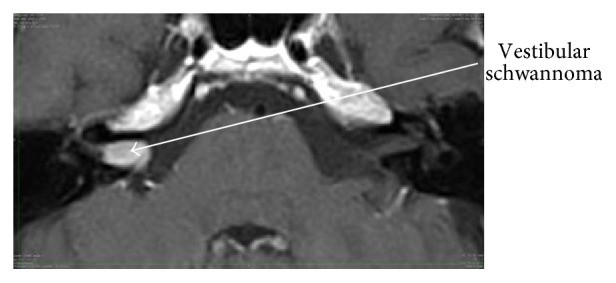
A vestibular schwannoma shown in a contrast-enhanced T1-weighted sequence.

**Figure 2:**
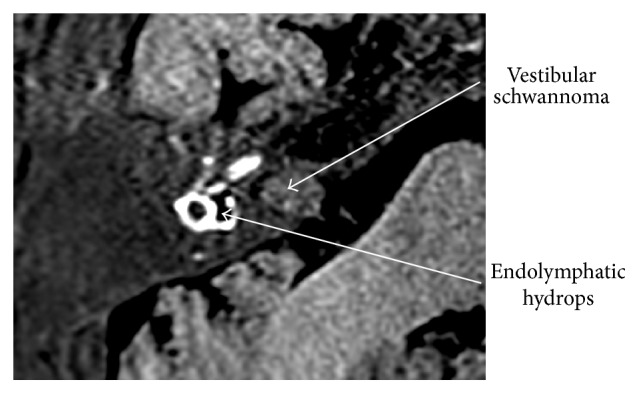
Endolymphatic hydrops and VS seen in a “locally enhanced inner ear MR imaging” (LEIM), 3D real inversion recovery MRI of the right inner ear after intratympanic Gadolinium contrast application. The vestibulum, the horizontal SCC and the basal turn of the cochlea are visible. Perilymph appears hyperintense, endolymph appears hypointense (black), and surrounding temporal bone appears grey. The vestibular endolymphatic space is clearly enlarged, indicating endolymphatic hydrops.

It is nowadays possible to demonstrate endolymphatic hydrops in vivo through MR imaging [56] and the degree of endolymphatic hydrops has been shown to correlate with loss of audiovestibular function in patients with Menière's disease [57–59]. MR imaging after local contrast application and image processing even enables the volumetric quantification of endolymphatic hydrops [60]. A classical picture of a VS on T1 weighted MRI after intravenous contrast application is shown in Figure 1.  Figure 2 demonstrates vestibular endolymphatic hydrops in a patient with a stable (over 7 years) small vestibular schwannoma who developed recurrent vertigo attacks during follow-up.

Naganawa et al. were able to identify vestibular endolymphatic spaces on noncontrast-enhanced 3D FLAIR (fluid attenuated inversion recovery) MRI. By using intrinsic perilymph signal increase in some patients with VS, endolymphatic hydrops was seen in four out of thirteen patients. The signal increase is attributed to the increased protein concentration [61]. In this small number of patients, there was no clear-cut correlation between symptoms and endolymphatic hydrops. However, the results suggest a surprisingly high prevalence of endolymphatic hydrops in VS patients and raise the possibility that, in analogy to Menière's disease, endolymphatic hydrops might contribute to audiovestibular function alterations in VS patients and this also raises the question whether specific therapeutic interventions directed against the endolymphatic hydrops might be useful, especially in patients during observational management of the VS.

It has been suggested that metabolic abnormalities caused by the tumour can have an important impact on the symptoms and that in this way abnormalities within the labyrinth may result in secondary injury to the organ of Corti. Thus, the progressive hearing loss could be caused by the release of toxins or potassium ions by the tumour. However, these metabolites have never been isolated or shown to be causative [62, 63].

The alteration of inner ear fluid composition in VS cases seems to play an important role. Different possible causes for this phenomenon have been described including impaired labyrinthine blood flow [64, 65], which may be induced, for example, by intralabyrinthine VS compression of the labyrinthine artery, cellular immune reactions [66–68], and/or blockage of neuroaxonal transport mechanisms [69]. Moreover, acidophilic-staining precipitate in a series of 21 sporadic VS cases has been found [70].

Finally, a small subset of patients may experience a mixed hearing loss, due to an inner ear conductive loss thought to be caused by interference with the intracochlear perilymphatic fluid wave or dampening of stapes movement [71, 72].

The different causes of labyrinthine damage by a VS are summarized as follows:

 Interference of microcirculation by nerve compression.
 Release of toxins or potassium ions by the tumour.
 Degeneration of the organ of Corti and the Stria vascularis.
 Changes of inner ear homoeostasis.
 Endolymphatic hydrops.

## 4. Conclusion

In order to preserve the auditory and vestibular function in VS patients, it is important not only to stop the growth of the tumour but also to avoid degenerative changes in the inner ear. A detailed neurotological workup helps in the diagnosis of VS of all sizes and can also provide useful prognostic information. In particular, caloric irrigation and unilateral hearing loss in audiometry have to draw the attention to the existence of a VS.

As false-negative results remain possible in small intrameatal tumours (1%) [73] MR imaging is advisable even if the above tests are negative; in addition, MRI data is required in patients with positive tests so that an appropriate treatment plan (surgical intervention or watch and wait therapy) can be produced. It remains questionable whether an MRI scan is considered urgent when audiovestibular tests show no pathologies.

Loss of vestibular function usually occurs slowly and gradually, allowing an effective central compensation, so vertigo symptoms are often absent until the loss of function is severe.

Moreover, extended clinical vestibular function tests including vHIT and VEMPs can determine the overall extent of vestibular function loss, especially because the traditional caloric irrigation can deliver normal results in VS patients.

It has been shown that there is a significant degeneration of labyrinthine structures in ears with VS. Nevertheless, it is still unclear whether VS symptoms like hearing loss and vertigo are caused primarily by these labyrinthine mechanisms or by retrocochlear mechanisms.

Traditionally, VS have often been regarded simply as retrocochlear lesions. However, otoacoustic emission recordings and, in recent years, the endolymphatic hydrops visualization clearly show significant secondary labyrinthine pathology in patients with VS. Further research, using this new diagnostic possibility and correlating the morphologic findings with audiovestibular function test results in patients with VS will likely provide new insights into the relative contributions of neural versus labyrinthine factors to the pathophysiology and symptoms of VS. Some symptoms may possibly arise from labyrinthine factors rather than from neural factors. Since the inner ear is better accessible to therapeutic interventions than the audiovestibular nerve, for example, by intratympanic application of drugs, new therapeutic possibilities for symptom control in VS patients may possibly arise in cases where the tumour is stable and does not require invasive interventions.

## Figures and Tables

**Table 1:** Tumour classification according to Koos et al. [24] and Samii and Matthies [25].

Grade (Koos et al.)	Grade (Samii and Matthies)	Definition of tumour size
I	T1	Purely intracanalicular lesion
II	T2	VS protruding into the cerebellopontine angle without brainstem contact
IIa	T2	Tumour diameter < 1 cm
IIB	T2	Tumour diameter 1–1,8 cm
III	T3a	Filling cerebellopontine angle cistern
	T3b	Reaching the brainstem
IV	T4a	Brain stem compression
	T4b	Severely dislocating the brainstem and compressing the fourth ventricle
